# Supplementary material for: Towards objective and systematic evaluation of bias in artificial intelligence for medical imaging
Source: J Am Med Inform Assoc. 2024 Jun 28;31(11):2613–21. doi: 10.1093/jamia/ocae165 (PMC11491635; doi:10.1093/jamia/ocae165)
Supplement: ocae165_Supplementary_Data [file ocae165_supplementary_data.pdf]

Supplementary Material for:

Towards objective and systematic evaluation of bias in medical imaging AI

Reweighing equations

The weight *W* for samples in disease class *D* and bias group *B* is given by:

$$W(B = b|D = d) = \frac{P_{exp}(b \wedge d)}{P_{act}(b \wedge d)}$$

Where:

$$P_{exp}(b \wedge d) = expected\ probability\ of\ an\ unbiased\ dataset.$$

And:

$$P_{act}(b \wedge d) = actual\ probability\ in\ dataset.$$

Thus, the weights for each class and bias group are:

$$W(B = 1|D = 1) = \frac{d(\gamma + \alpha)}{\gamma}$$

$$W(B = 1|D = 0) = \frac{(1 - d)(\gamma + \alpha)}{\alpha}$$

$$W(B = 0|D = 1) = \frac{d(2 - \gamma - \alpha)}{1 - \gamma}$$

$$W(B = 0|D = 0) = \frac{(1 - d)(2 - \gamma - \alpha)}{(1 - \alpha)}$$

Where:

$$\alpha = fraction\ of\ bias\ group\ in\ non\text{-}disease\ class.$$

$$\gamma = fraction\ of\ bias\ group\ in\ disease\ class.$$

$$d = fraction\ of\ disease\ class\ in\ full\ dataset.$$

Supplementary Tables

Table S1: SimBA effect sampling parameters.

	Subject	Disease Class	Non-Disease Class	Bias Group	Non-Bias Group
Number of Samples	2002	1000	1002	1003	999
Region	Whole brain	Left insular cortex	Left insular cortex	Near: Left putamen<br>Far: Right postcentral gyrus	-
Sampling Distribution	N(0,1) ∈ [-3.5,3.5]	N(-1,1)	N(1,1)	N(2,1)	-
Number of Principal Components Sampled From	10	1	1	1	-
Number of Stratification Bins	10	10	10	-	-

Table S2: Composition of dataset splits.

	Train		Validation		Test	
	Disease	Non-Disease	Disease	Non-Disease	Disease	Non-Disease
Non-Bias	140	360	72	176	68	183
Bias	360	145	180	70	180	68

Table S3: True positive rate statistical tests. Bold font indicates groups in comparison test. Two-tailed paired t-test unless indicated by *, then Wilcoxon matched-pairs signed rank test. †significant after Bonferroni correction (α=0.005).

Model	Bias Scenario	P-value
Naïve	**No Bias – Near Bias**	<0.0001†
Naïve	**No Bias – Far Bias**	0.0002†
Naïve	**Near Bias – Far Bias**	0.1037
**Naïve – Reweighing**	Near Bias	0.0001†
**Naïve – Unlearning**	Near Bias	0.0075
**Naïve – Group Models**	Near Bias	0.8534
**Naïve – Reweighing***	Far Bias	0.0625
**Naïve – Unlearning**	Far Bias	0.0104
**Naïve – Group Models***	Far Bias	0.0625
**Naïve – Group Models**	No Bias	0.0007†

Table S4: False positive rate statistical tests. Bold font indicates groups in comparison test. Two-tailed paired t-test unless indicated by *, then Wilcoxon matched-pairs signed rank test. †significant after Bonferroni correction (α=0.005).

Model	Bias Scenario	P-value
Naïve	**No Bias – Near Bias**	<0.0001†
Naïve	**No Bias – Far Bias**	0.0008†
Naïve	**Near Bias – Far Bias**	0.0552
**Naïve – Reweighing**	Near Bias	0.0002†
**Naïve – Unlearning**	Near Bias	0.0525
**Naïve – Group Models**	Near Bias	0.6071
**Naïve – Reweighing**	Far Bias	0.0058
**Naïve – Unlearning***	Far Bias	0.0625
**Naïve – Group Models**	Far Bias	0.0289
**Naïve – Group Models**	No Bias	0.0001†

Table S5: Results of bias group prediction after unlearning

Bias Scenario	Bias Prediction Accuracy (%)
No Bias	49.94 ± 0.33
Near Bias	47.17 ± 4.23
Far Bias	46.73 ± 7.98

Table S6: Weighted saliency scores (%) for the Near Bias region across five model weight initialization seeds.

		Disease Class		Non-Disease Class	
Bias Scenario	Bias Mitigation Strategy	Non-Bias Group	Bias Group	Non-Bias Group	Bias Group
No Bias	Naïve	47.32 ± 7.23	47.18 ± 8.35	30.34 ± 7.55	30.55 ± 6.85
	Reweighing	40.97 ± 4.37	39.88 ± 4.73	23.25 ± 5.97	23.48 ± 5.62
	Unlearning	46.14 ± 7.66	44.98 ± 6.86	30.67 ± 6.84	29.52 ± 6.51
	Group Models	46.91 ± 1.40	45.92 ± 6.64	30.76 ± 7.95	29.53 ± 8.58
Near Bias	Naïve	77.09 ± 12.11	75.68 ± 14.42	45.78 ± 7.01	55.81 ± 16.84
	Reweighing	41.76 ± 4.45	42.7 ± 3.89	19.22 ± 5.68	20.51 ± 5.52
	Unlearning	59.64 ± 13.28	54.97 ± 9.37	36.02 ± 12.65	37.34 ± 13.98
	Group Models	42.15 ± 4.03	39.28 ± 6.95	27.1 ± 4.92	21.59 ± 4.70

Table S6: Weighted saliency scores (%) for the Far Bias region across five model weight initialization seeds.

		Disease Class		Non-Disease Class	
Bias Scenario	Bias Mitigation Strategy	Non-Bias Group	Bias Group	Non-Bias Group	Bias Group
No Bias	Naïve	1.69 ± 0.76	1.67 ± 0.72	1.22 ± 0.42	1.14 ± 0.32
	Reweighing	1.34 ± 0.70	1.31 ± 0.69	1.35 ± 0.67	1.34 ± 0.67
	Unlearning	1.13 ± 0.52	1.12 ± 0.53	0.98 ± 0.25	0.95 ± 0.28
	Group Models	1.91 ± 0.99	1.5 ± 0.55	1.59 ± 1.00	1.41 ± 0.63
Far Bias	Naïve	7.77 ± 3.35	7.85 ± 3.32	7.47 ± 3.56	7.39 ± 3.55
	Reweighing	2.23 ± 0.79	2.09 ± 0.66	1.48 ± 0.70	1.48 ± 0.70
	Unlearning	10.47 ± 6.04	10.41 ± 6.16	8.66 ± 4.25	8.65 ± 4.46
	Group Models	1.91 ± 0.60	3.12 ± 1.53	1.6 ± 0.49	2.64 ± 1.70

Supplementary Figures

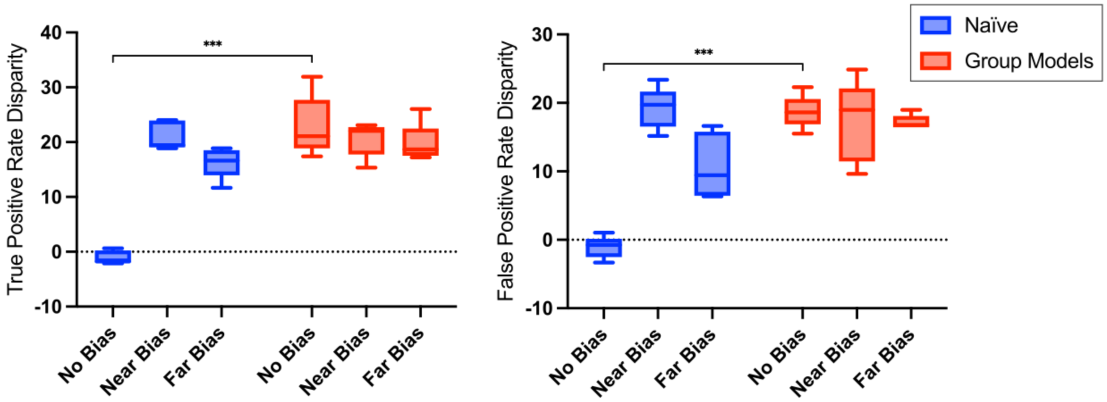

Figure S1: True (left) and false (right) positive rate disparities for the naïve models and group models. ***p<0.001.
